# Oral nutritional supplements for older hip fracture patients at nutritional risk (NUTRI-MUSCLES)—a feasibility trial for a two-armed RCT

**DOI:** 10.1186/s40814-026-01763-4

**Published:** 2026-01-21

**Authors:** A. Jensen, E. D. Ninh, I. Tetens, A. M. Beck

**Affiliations:** 1 Research Unit for Dieticians and Nutrition Research, Herlev Hospital, Borgmester Ib Juuls Vej 1, 20Th Floor, Herlev, 2730 Denmark; 2 Department of Nutritional, Exercise and Sports, University of Copenhagen, Copenhagen, Denmark

## Abstract

**Background:**

Malnutrition is prevalent in older hip fracture patients and increases the risk of postoperative complications and loss of physical function. Adequate energy and protein intake during rehabilitation can enhance recovery but treatment can be challenging. The primary objective was to assess feasibility, defined as eligibility, recruitment rate, retention in the study, compliance to the intervention and completeness of outcome data collection.

**Methods:**

This single-site, open labelled, parallel, two-arm randomized controlled feasibility trial took place at Orthopaedic Surgical ward at Herlev Hospital, Denmark. Participants were ≥ 65 years at nutritional risk admitted with a hip fracture. The intervention group received two bottles of oral nutritional supplements daily for 12 weeks after discharge. The control group received standard care.

**Results:**

Of 134 patients screened, 52 (39%) met inclusion criteria and 21 (40%) were recruited, corresponding to 2.3 participants per week. Preliminary findings showed a retention rate of 16 out of 21 (76%). Compliance to the oral nutritional supplements was 62% reflecting an intake of 1.3 oral nutritional supplementation per day. Data collection was high, with ≥ 80% completeness for most outcomes (handgrip strength, calf circumference, frailty, quality of life, activities of daily living, 24-h recall, blood measures), except for the baseline 30-s chair-stand test.

**Conclusion:**

The trial methods were feasible with sufficient eligibility, recruitment rate, retention in the study and outcome data collection. Compliance was lower than expected highlighting the need for strategies to improve adherence in the definitive trial.

**Trials registration:**

ClinicalTrials.gov identifier: NCT05556876. Date of registration: 2022.23.09. URL: https://clinicaltrials.gov/study/NCT05556876.

**Supplementary Information:**

The online version contains supplementary material available at 10.1186/s40814-026-01763-4.

## Key messages regarding feasibility

- What uncertainties existed regarding the feasibility?
  Uncertainties regarding a sufficient recruitment, retention rate, compliance and completeness of outcome data collection remained due to older surgical patients with frailty and fast track surgery with shortened hospital stays.
- What are the key feasibility findings?
  The eligibility, recruitment rate, retention in the study and completeness of outcome measures was feasible. Compliance was lower than expected.
- What are the implications of the feasibility findings for the design of the main study?
  The findings of this feasibility study provide important information about the necessity of implementing measures to improve compliance towards the intervention.

## Background

Hip fractures are the most common orthopaedic injury among older adults, with 7702 cases reported in Denmark in 2022 [1, 2]. As life expectancy increases, the incidence of hip fractures in older patients is expected to rise, increasing the burden on healthcare systems and the treatment of these patients [1]. Sustaining a hip fracture leads to hospitalisation, immobility and further decline in muscle mass and function, significantly affecting both immediate and long-term health outcomes [1]. Only 40–60% of older adults regain their pre-fracture mobility, and many experience higher mortality rates, post-operative complications and reduced quality of life (QoL) [3–7].

Furthermore, malnutrition is prevalent among older hip fracture patients and a systematic review revealed that 35.3% of older hip fracture patients admitted to hospital were at risk of malnutrition and 18.7% were already malnourished at the time of admission [8]. Malnutrition increases the risk of postoperative complications including infections, impaired wound healing, risk of pressure ulcers, impaired functional ability, longer duration of hospitalisation, re-admissions and mortality [9]. Addressing malnutrition through adequate energy and protein intake can enhance recovery and physical function during rehabilitation [4]. However, the majority of previous studies were performed a long time ago and does not reflect the present population with very old and frail surgical patients [2]. Further the surgical treatment and follow-up used today have changed with the introduction of fast-track surgery and shortened length of hospital stays [10]. This means that the beneficial results obtained in the older studies might not be reproducible to today. In addition to this, critical outcome measures such as nutritional and hydration status, QoL and functional performance are underrepresented in former studies. These outcomes have been identified as critical in evaluating the efficacy of nutritional interventions in older adults who are malnourished or at risk of malnutrition [11]. Hence, there is a need for intervention studies reflecting the present population, the novel treatment procedures and including critical outcome measures.

Before the initiation of intervention studies, it is relevant to consider whether it is possible to recruit and retain the old patients, since both are known challenges [12]. Specifically due to the increasing age, many old patients may not be eligible for the inclusion due to for example cognitive impairment. Further, the shortened length of stay can make it difficult to obtain the informed consent to participate. The use of oral nutritional supplementation (ONS) is recommended for older hip fracture patient to provide sufficient energy and protein intake [4]. However, previous studies have shown that compliance to ONS can be challenging especially among the older adults [13]. Finally, due to the frail population, patient-centred outcomes such as physical function and QoL may be difficult to obtain. Therefore, the primary objective of the study was to assess feasibility defined as eligibility, recruitment rate, retention in the study, compliance to the intervention and completeness of outcome data collection. These findings will inform the development of a future definitive RCT where the primary objective is to assess the effect of post-discharge ONS in older patients operated for hip fracture.

## Methods

The trial was designed as a single-site, open labelled, parallel randomized controlled feasibility trial with two arms. Participants were randomized in block sizes of 2, 4, 6 and 8 by computer-generated randomization in a 1:1 ratio. Masking of the allocation was ensured by an impartial staff member with no clinical involvement in the trial, who prepared the allocation sequence and the sealed envelopes. A total of 130 envelopes containing a piece of paper with the allocated group A or B were made ready. Concealment was ensured by wrapping the paper in aluminum foil to render the envelopes impermeable in intense light. The envelopes were stored in an enclosed cupboard in a locked room only accessible to research staff. After the collection of baseline data, the subsequent envelope was delivered to the participant by investigators to reveal the designated allocation. To ensure consistency in data collection, standard operational procedures for each outcome were prepared in advance. The study is presented in accordance with the CONSORT guidelines for randomized pilot and feasibility studies [14]. For the CONSORT checklist see additional file 1.

### Participants

The inclusion for this feasibility study began on the 4th of December 2023 and the final 12-week follow-up was performed on 7th of May 2024. Eligible participants were ≥ 65 years, at nutritional risk according to the Nutritional Risk Screening 2002 (NRS-2002) [15], admitted with a hip fracture to the Orthopaedic Surgical ward at Herlev Hospital, Denmark, and expected to be discharged with a rehabilitation plan. Hip fracture types included femoral neck fracture, pertrochanteric fracture or subtrochanteric fracture. Exclusion criteria included active cancer, renal inefficiency (eGFR < 27, mL/min/1.73 m^2^), cognitive impairment (inability to comprehend the trial purpose or give informed consent), terminal illness, exclusive reliance on texture-modified food, allergies to milk or fish, enteral or parenteral nutrition, intention to lose weight or go on a special diet, current use of fish oil supplements for medical reasons such as hypertriglyceridemia, unwillingness to discontinue fish-oil supplements during the trial period or inability to understand or speak Danish. The cognitive status was systematically approached by screening for diagnosed Alzheimer’s or dementia registered in the Danish Health Informatics platform. Prescription of other ONS at discharge were not an exclusion criterion. Participants in the intervention group who were prescribed additional ONS were instructed to prioritize the study provided ONS and either use prescribed ONS as a supplement or save the prescription until after the study period. In the control group, any ONS prescription was recorded. The prevalence of such prescriptions was expected to be low [16].

Primary screening of all patients admitted to the orthopaedic surgical ward was done by a daily inventory of patients ≥ 65 years old admitted with a hip fracture. This was facilitated through the Danish Health Informatics platform. Secondary screening and initial contact were carried out by the clinical dietitians on the project. Eligible patients received both written and oral information before signing an informed consent form.

### Intervention

**Fig. 1:**
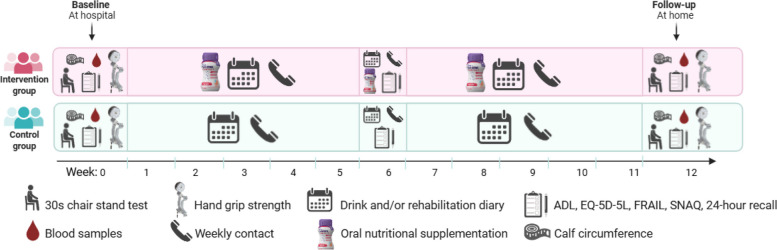
Trial design

An overview of the trial design is presented in Fig. 1. The intervention group was provided with the study ONS (S-ONS) which should be ingested from the first day of discharge from hospital and continued for 12 weeks. Details about the nutritional content can be found in Table 1. Two different flavours (red berries or peach/mango) were available of the S-ONS. Before discharge, the participants received counselling by study investigators to drink a total of 250 mL per day corresponding to two servings at times most suitable for them. Participants were also provided with written material on serving recommendations and the importance of protein intake. The S-ONS was provided free of charge by Danone. At discharge, the participants were given a 1-week supply of S-ONS. During the 12-week intervention period, S-ONS were delivered to the participants home twice and adjustments in flavour preferences were done based on feed-back from the participants (see details below).

**Table 1:** Nutritional content in the S-ONS provided to the intervention group

Content	100 mL	250 mL
Energy (kcal)	245	613
Protein (g)	15	37
Fat (g)	9.6	24
Omega-3 (g)	1.5	3.7
Of which EPA (g)	0.9	2.2
Of which DHA (g)	0.6	1.5
Carbohydrate (g)	25	63
Vitamin D (µg)	8	20

### Daily drink diary

After discharge, the intake of S-ONS was assessed by a ‘Daily drink diary’. The participants were asked to daily register the amount of beverage consumed; 0%, 25%, 50%, 75% or 100% of each of the two bottles prescribed per day. Participants received weekly phone calls by study investigators, reminding them about consuming the supplement, complete the diary and preferences for flavour. Compliance was evaluated by the daily drink diary and by counting remaining cans. Compliance (%) was calculated as (number of supplements consumed/number prescribed) × 100. When both can-count and diary compliance values were available for a participant, the mean of the two percentages was used as the overall compliance. If only one source was available, that value was used. High compliance was defined as an average consumption of ≥ 75% of the total prescribed S-ONS [17]. Participants were given a folder to note any side-effect potentially related to the S-ONS. Side-effects mentioned during weekly phone calls were documented.

### Rehabilitation care and rehabilitation diary

Both the intervention and the control group were prescribed post-discharge rehabilitation as part of standard care. These rehabilitation plans did not specify duration or intensity, as implementation was determined by individual municipalities, leading to variability across participants. Completion of rehabilitation was assessed through self-report, a method deemed feasible in prior studies [18]. Participants were also asked to document any additional physical exercise. Weekly follow-up phone calls were conducted by investigators to monitor rehabilitation progress and remind participants to record their activities in a rehabilitation diary. The rehabilitation diary data were not included in this feasibility study.

### Outcomes

Data was collected at baseline and at 12-week follow-up. Additionally, physical function and quality of life questionnaires were assessed at 6-week follow-up. Only the baseline and 12-week follow-up data are presented in this article.

### Primary outcome

#### Feasibility of trial methods

The primary objectives of the study were to assess feasibility defined as eligibility, recruitment rate, retention in the study, compliance to the intervention and completeness of outcome data collection.

#### Eligibility

To assess eligibility, data was collected on the number of patients who met the inclusion criteria. According to the Danish Hip Fracture registry, almost all of the around 800 patients operated per year for femoral neck, pertrochanteric or subtrochanteric fractures at our hospital are prescribed a rehabilitation plan at discharge [19]. Nutritional risk was assessed using the validated screening tool Nutritional Risk Screening 2002 (NRS-2002), as recommended by the Danish Health Authority [15]. Hip fracture patients were assigned a minimum of 2 points for severity of illness due to surgery and immobilization along with at least 1 point for nutritional status, reflecting the anticipated reduction in dietary intake [15]. This results in all hip fracture patients being categorized at nutritional risk with a minimum score of 3. Hip fracture patients aged 70 and above receive an additional point, resulting in total score of 4. Based on these criteria, it was expected that 100% of the hip fracture patients with femoral neck, pertrochanteric or subtrochanteric fractures would be eligible for this study.

#### Recruitment and retention

The recruitment rate was assessed based on number of included participants per week. Retention rate was calculated as the proportion of included patients who completed the 12-week intervention period. Based on previous similar studies, the expected recruitment rate was three participants per week and the anticipated drop-out rate was 30% [17, 20]. Hence, a retention rate of 70% was expected.

#### Compliance to the intervention

The S-ONS used in our study has not been provided to patents with hip fracture before and the expected compliance is unknown. In a previous study, patients with cancer have been provided with two servings of the S-ONS for 12 weeks, reported an average consumption of 1.7 bottles per day (85%) [21]. Hence, this was also what we expected. In addition, we expected to see a difference in the total intake of energy and protein among the intervention compared to the control group due to the extra provision of energy and protein of, respectively 613 kcal and 37 g protein provided from the S-ONS.

#### Completeness of outcome data collection

Feasibility of the trial methods was further evaluated by assessing completeness of the secondary outcome measures (see details below). They were considered feasible if the data collection was ≥ 80% [22].

### Secondary outcomes

All secondary outcomes were collected by trained study investigators. It was attempted that the same investigator performed all the data collection in one patient to reduce the risk of performance bias.

### Questionnaires

#### Energy and protein intake

The participants’ energy and protein intake were measured through a single 24-h dietary recall questionnaire at 12-week follow-up. The aim was to compare intake between the groups. Further, the results from the dietary recall were used to evaluate the compliance in the intervention group and assess if the intervention was successfully implemented. Participants were instructed to give a detailed description of a typical daily dietary intake. In both groups, there was a specific focus on the intake of ONS, either the S-ONS or other types. Data from the interview was recorded in a standardised sheet. The standardised sheet also sufficed as a guiding tool throughout the interview. A picture chart developed by the Technical University of Denmark was used to help determine portion sizes [23]. The dietary intake was reported as detailed as possible, including type of product (light, neutral, wholegrain), brands, fat content, sugar or sugar free, fibre etc. The interviewer was sometimes able to inspect refrigerators to determine the precise food consumed.

The intake of energy and protein was calculated by means of the Danish food database © 2025 VITAKOST. The database provided comprehensive nutritional information for a wide range of food items, which allowed for precise analysis of nutrient consumption. Cut-off for underreporting was set at 1.1 ratio calculated from energy intake and basal metabolic rate [24]. Results were reported for the total intake of energy and protein.

#### Physical function

The Functional Recovery score (FRS) was used to assess restoration of function to pre-fracture level. The FRS is self-reported and thus based on participants self-evaluation. Research assistants interviewed the participants using the scoring guidance to assure a thorough reporting of the participants’ functions. At baseline, the participants were instructed to answer based on the pre-fracture function, while at 12-week follow-up they answered based on their current function. Results were reported as total Activities of daily living (ADL), Basal activities of daily living (BADL), Instrumental activities of daily living (IADL), mobility score and the proportion of participants who regained their pre-fracture physical function.

#### Frailty

The FRAIL scale was used to evaluate the level of frailty in the participants. It has been designed for clinical application to help identify frailty-related issues in older adults [25]. The self-reported FRAIL scale questionnaire identifies the phenotype of frailty on the following 5 components: fatigue, resistance, ambulation, illness and loss of weight. The total score ranges from 0 to 5, where 1 point can be given for each component [26]. Frailty was indicated by the presence of 3 or more items (3–5 points), while pre-frailty was indicated by the presence of 1 or 2 items (1–2 points). When none of the items were present (0 points), the person had a robust health condition [26]. Pre-frailty indicated a risk of rapid progression into the frail stage [25]. The results were reported as number of participants categorized as frail or pre-frail.

#### Health-related quality of life

The European Quality of life-5 Dimensions-5 Levels (EQ-5D-5L) was used to assess health-related quality of life (HRQoL). The questionnaire is self-reported and contains two parts [27]. The first part consists of 5 questions (dimensions) concerning mobility, self-care, usual activities, pain/discomfort and anxiety/depression. Each question has 5 response categories (levels), ranging from having no problems to extreme problems/being unable [27]. The responses imply a health state, which is converted into an index score (utility), to reflect how good or bad the health state is compared to the general population. Danish population norm data for the EQ-5D-5L were used, where an index score of − 0.757 is the worst possible health status and 1.000 indicates maximum health [28, 29]. An index score below zero is considered worse than being dead [28]. The second part of the questionnaire is a visual analogue scale (EQ-VAS) ranging from 0 (the worst health you can imagine) to 100 (the best health you can imagine) [27]. A license to use the official Danish questionnaire was obtained. Participants were interviewed in the EQ-5D-5L questionnaire by the research assistants. The participants were asked to subjectively report the degree of problems they experienced within each of the 5 questions. Using the VAS-scale, participants were asked to summarise their current overall health condition within the range of the scale. The results were reported as the index score and VAS score.

#### Appetite

The Simplified Nutritional Appetite Questionnaire (SNAQ) investigates appetite based on four questions. It assesses self-reported appetite, taste, satiety following meals and usual meal frequency [30]. Each question is scored from 1 to 5, resulting in a total score from 4 to 20. The SNAQ can identify individuals with anorexia of ageing, who are at risk of.

developing weight loss over the next 6 months [30]. A total score of ≤ 14 can predict a 5–10% weight loss [31]. Results were reported as the total score and as the proportion of participants with a total score of ≤ 14.

## Measurements

### Blood samples

Non-fasting venous blood samples were taken during hospitalisation and at 12-week follow-up. Blood samples were analysed for vitamin D (25-OH-Vitamin D), albumin, sodium, potassium, glucose, urea and CRP. The blood sample were collected at time of admission to the emergency medical department, except for CRP which was the result closest to discharge. The research assistants were trained in performing blood samples and collected a total amount of 7 mL at the 12-week follow-up visit. Blood samples were analysed at the clinical Biochemistry Department at Herlev Hospital, Denmark, and retrieved from the electronic patient record.

Simple hyperosmolar dehydration was calculated using the Eq. (1.86 × (Na + + K +) + 1.15 glucose + urea + 14) [4]. The cut-off value for determining low-intake dehydration was ≥ 295 mOsm/L. Patients were categorized as normohydrated if the calculated osmolarity was < 295. Calculated osmolarity has been validated in older hospitalized medical patients [32, 33]. The results were reported as the calculated osmolarity and the proportion of participants categorized as dehydrated.

Omega-3 was assessed by a finger prick test. The analysis provides omega-3 index, omega-6/omega-3 ratio and content of eicosapentaenoic acid (EPA) and docosahexaenoic acid (DHA) fatty acids.

### Muscle mass

Muscle mass was estimated by measuring calf circumference on the non-operated leg. A

calf circumference below 33 cm for men and 32 cm for women implies a low skeletal muscle mass [34, 35]. When performing the measurement, the participant should be in a seated position with the legs resting on the floor and the trouser leg rolled up. Using a measurement tape, the calf circumference was measured at its greatest point without pressing the skin. Several repeated measurements were done, assuring that the greatest point was recorded. If the participant was in bed, the measurement was done with the leg bent and the foot resting on the mattress [36]. Results were reported as median calf circumference in cm and number of participants categorized with a low muscle mass.

### Muscle strength

The 30-s chair-stand-test (30 s-CST) was used to measure muscle strength in.

the lower extremities. The number of times the participants could stand and sit from a standard chair within 30 s was counted and recorded as the result [37, 38]. The chair height should be between 44 and 47 cm [39]. In the standard version, the arms must be folded across the chest, while in the modified version the use of armrest was allowed. Results were reported as number of repetitions for the standard and modified version separately, and the percentage of participants performing the standard version of the test was reported. When participants were unable to perform a version of the test, they scored zero.

Hand grip strength (HGS) measured in kg was used as a proxy for muscle strength of the upper body extremities. HGS was measured in the dominant hand by the Saehan DHD-1 Digital Hand Dynamometer. The measurement was performed based on a procedure for HGS measurement developed at Department of Physio- and Occupational-therapy at Bispebjerg-Frederiksberg Hospital [40]. The test was repeated 3 times with 15 s breaks in between. The highest score was documented for the results. Results were reported in kilograms.

### Sample size justification

No formal sample size calculation was performed for this trial, as the primary outcome measure were to assess eligibility, recruitment rate, retention and the feasibility of the secondary outcome measures [41]. The target sample size for the feasibility study was approximately 20–25 participants, corresponding to three participants per week, and the number of participants that could be recruited during a predefined 2-month inclusion period. This sample size was considered sufficient to estimate key feasibility parameters, including recruitment and retention rates, intervention compliance and data completeness, with adequate precision to inform the design of a future definitive randomized controlled trial [42]. The sample size for the future definitive RCT was calculated to be 124, based on a clinically relevant difference in the primary outcome 30 s-CST, SDs reported in similar patient groups and an expected drop out of 30% [13, 37, 43]. Hence, these key study parameters were essential to investigate in the present feasibility study.

### Feasibility success criteria

Progression criteria were established to determine whether it would be appropriate to proceed to a definitive randomized controlled trial. These criteria were based on feasibility objectives and informed by previous trials in similar patient populations and published guidance for feasibility studies.

Progression criteria included achieving eligibility and recruitment rates sufficient to support timely recruitment in a future definitive trial (tree participants per week) and acceptable participant retention (70%) throughout the follow-up period. Feasibility was considered successful if the predefined criteria were met overall.

### Statistical analysis

The statistical analysis was performed using Statistical Analysis System (SAS), version 8. The feasibility outcomes were reported using narrative and descriptive statistics. The secondary outcomes were reported using descriptive statistics, median (IQR) for continuous outcomes and count (%) for categorical outcomes.

## Results

### Participants characteristics

Baseline characteristics for 21 randomized participants are presented in Table 2

**Table 2:** Baseline characteristics for included participants

	**Intervention (*n* = 10)**	**Control (*n* = 11)**	**All (** ***n*= 21)**
**Age, years**	80 (76–86)	87 (75–91)	84 (76–90)
Gender, female *n* (%)	4 (40)	5 (45)	9 (43)
Height, cm	173 (167–185)	169 (163–176)	173 (167–179)
BMI, kg/m^2^	24.6 (24.0–24.6)	22.1 (21.0–24.5)	24.0 (21.8–25.2)
**Muscle strength**			
30 s-CST, modified, n (%)	3 (30)	1 (9)	4 (19)
Hand grip strength, kg	18.7 (15.7–31.0)	23.7 (14.7–18.4)	18.8 (15.7–30.5)
**Muscle mass**			
Calf circumference, cm	35.6 (33.5–36.5)	32.0 (31.0–35.0)	34.0 (32.0–36.0)
**Physical function, ADL (0–100)**^**a**^	96 (79–100)	99 (82–100)	99 (80–100)
**Frailty, *n* (%)**			
Frail	4 (40)	5 (45)	9 (43)
Pre frail	6 (50)	6 (55)	12
**Health-related quality of life**			
EQ-5D-5L index (− 0.757–1.000)	0.410 (0.017–0.755)	0.178 (− 0.074–0.467)	0.232 (0.010–0.477)
EQ-5D-5L VAS (0–100)	55 (40–78)	40 (30–60)	50 (35–60)
**Biomarkers**			
Vitamin D, nmol/L	77 (57–95)	93 (76–122)	91 (64–104)
Albumin, g/L	25.5 (23.0–30.0)	26.0 (22.0–30.0)	26.0 (23.0–30.0)
Osmolarity, mmol/L	297 (295–298)	296 (294–298)	297 (294–298)
Dehydrated, *n *(%)	7 (70)	6 (55)	13
CRP, mg/L	109 (67–185)	96 (49–197)	96 (67–187)
Omega-3 index, %	6 (5–6	6 (5–8)	6 (5–7)
Omega-6/Omega-3 ratio	10 (9–11)	9 (4–13)	9 (7–11)
EPA, %	0.9 (0.8–1.4)	1.0 (0.7–1.7)	1.0 (0.8–1.5)
DHA, %	3.3 (3.0–3.7)	3.5 (2.3–4	3.4 (2.6–3.8)
**Appetite**			
SNAQ ^b^	15 (13–17)	15 (13–16)	15 (13–16)
SNAQ score ≤ 14, *n* (%)	5 (50)	4 (36)	9 (43)
**Discharge destination, *n* (%)**			
Rehabilitation center	7 (70)	8 (73)	15
Home	3 (30)	3 (27)	6 (29)
**Length of hospital stay, days**	7 (4-8)	8 (6-8)	8 (5-8)

Data are presented as median (IQR) for continuous variables and as number of individuals (percentage) for categorical variables. *ADL* activities of daily living, *BMI* body mass index, *CRP* C-reactive protein, *HGS* hand grip strength, *SNAQ* simplified nutritional appetite questionnaire, 30 s*-CST* 30 s chair stand test

^a^ADL pre fracture

^b^SNAQ scores between 5 and 20

## Primary outcome

### Feasibility of trial methods

#### Eligibility, recruitment and retention

During the study period, 134 patients aged ≥ 65 years were at nutritional risk and expected to be discharged with a rehabilitation plan; these patients were screened for eligibility. Of these, 52 (39%) met the inclusion criteria, and 21 (40%) were included in the study, resulting in a recruitment rate of 2.3 participants per week. Reasons for non-participation included, they found it too demanding (*n* = 14), dislike of S-ONS (*n* = 7), feeling overwhelmed (*n* = 9) and in-hospital death (*n* = 1).

**Fig. 2:**
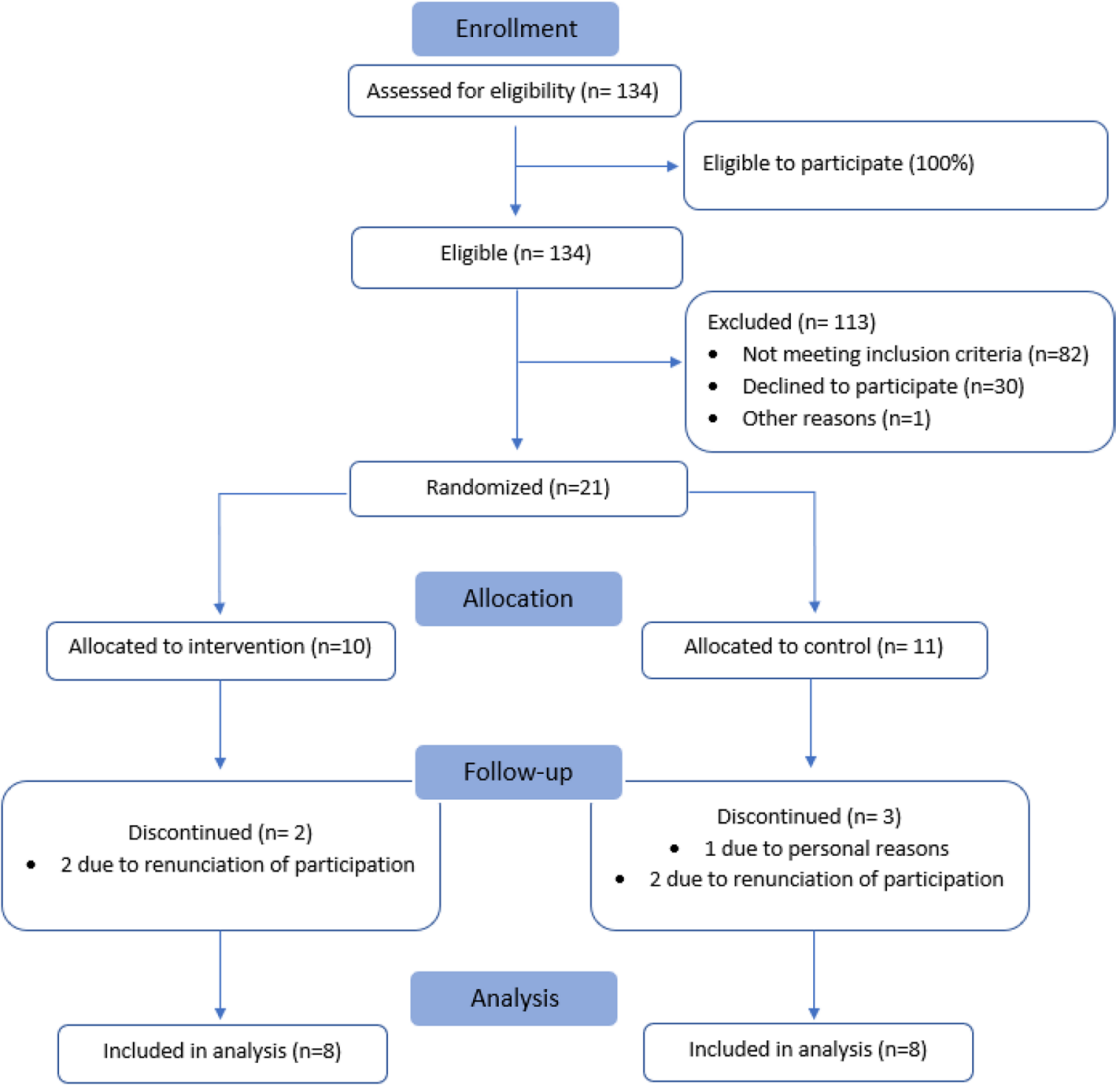
CONSORT flow diagram. Flow-chart showing inclusion, randomization and participation throughout the study

Allocation resulted in 10 participants in the intervention groups and 11 in the control group. Findings showed a retention rate of 16 out of 21 (76%). A total of two and three participants withdrew from the intervention and control group respectively, corresponding to a drop-out rate of 24%. Thus, at follow-up, 8 participants remained in each group. Reasons for withdrawal included regretting participation and feeling overwhelmed by their current situation. Figure 2 presents the flow-chart for the feasibility study, including inclusion, randomization and participation throughout the study period.

#### Daily drink dairy

A total of 5 out of 8 participants returned their daily drink diary at follow-up. Reasons for not returning included loss of diary, or failure to record intake. The participants who did not record in the diary either stopped because they forgot it or because they stopped consuming the drinks. Missing data from the diaries were accounted for by counting cans.

#### Compliance to the intervention

Compliance to the S-ONS was 61% calculated by counting cans and 79% by daily drink diary. The compliance calculated from the mean of the two percentages was 62% reflecting an intake of 1.3 S-ONS per day. Four out of eight had a compliance ≥ 75%.

**Fig. 3:**
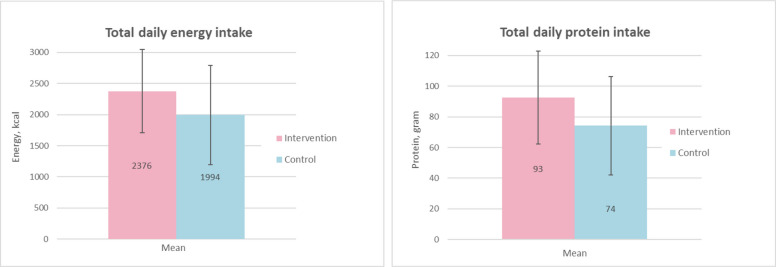
Mean intake of energy and protein at 12-week. Vertical lines shows the mean and SD of energy and protein intake

Three participants in the intervention group reported minor adverse events, including decreased appetite, nausea and weight gain. Two of these participants discontinued the use of S-ONS due these events. Three participants in the control group consumed other types of ONS. The result on energy and protein intake from the 12-week follow-up with and without the contribution from the S-ONS can be found in Fig. 3. The intervention group consumed an average of additional 382 [95% CI: − 484 to 1247] kcal and 18 [95% CI: − 15 to 52] g of protein compared to the control group.

#### Completeness of outcome data collection

At baseline, 4 out of 21 could perform the 30 s CST (using armrests). Participants who did not perform the 30 s CST test were bedridden during the baseline visit and unable to mobilize to a chair or stand without assistance beyond using the armrests. Other reasons for not performing the test included pain and lack of motivation. Except for baseline 30 s CST, all outcome measurements had a completeness of ≥ 80%.

The collection of outcome data at week 12 was assessed for 16 participants. Follow-up data showed that 15 out of 16 participants could perform the 30 s CST at 12 weeks post-discharge, with 11 out of 16 completing the standard version without using armrests. The one participant who could not perform the test was wheelchair bound and unable to stand.

Blood samples were obtained for 20 out of 24. Two of the blood samples was unsuccessful due to difficulties identifying a vein, while another was not collected because the participant declined an additional attempt. Results from the fourth blood sample were missing due to error in the laboratory analysis. Collection of all remaining outcome during the 12-week follow-up were completed successfully with a completeness rate of ≥ 80%.

## Discussion

The primary objective of this study was to assess feasibility defined as eligibility, recruitment rate, retention in the study, compliance to the intervention and completeness of outcome data collection.

### Eligibility, recruitment and retention

In this study, feasibility success criteria were predefined based on results from comparable previous trials, with expectations regarding recruitment, inclusion and retention. The percentage of eligible participants was high, as expected based on the selected inclusion criteria. However, the recruitment rate of 2.3 participants per week was lower than expected compared with former studies. Reasons for the lower recruitment rate was likely due to the inclusion of surgical patients, who are often more frail and therefore more likely to decline participation. To improve recruitment, the inclusion criteria were broadened to include other types of hip fractures and associated diagnostic codes related to complications from fractures in the hip and lower extremities where patients have a similar treatment and rehabilitation trajectory. These included sequela fracture of femur or distal femur fractures. A broader set of inclusion criteria will improve the external validity of the study results. Furthermore, we do not expect that the changes will reduce the comparability since the treatment and rehabilitation are comparable for these different types of fractures.

The retention rate was higher than expected, resulting in a lower drop-out rate. This may influence the sample size needed for the future definitive RCT. Although a drop-out of 30% was assumed, no adjustment has been made to the sample size at this stage due to the relative low number of the participants in the present study.

The anticipated inclusion rate was not fully achieved. In contrast, retention exceeded expectations, resulting in a lower drop-out rate than projected. Overall, the balance between lower-than-expected inclusion and higher-than-expected retention met our predefined feasibility criteria and supports the decision to proceed with a definitive randomized controlled trial.

### Barriers to intervention and measures to enhance adherence

Compliance with the S-ONS was lower than expected, which may affect muscle strength, the primary outcome in the definitive RCT. The average intake of S-ONS was 1.3 bottles per day corresponding to a mean compliance of 62%. Despite the low compliance, the difference in energy and protein intake was 382 g and 18 g, respectively. A reason for the low compliance could be that only two different flavours of the S-ONS were available, as more choices of flavours can increase the compliance [13]. Several participants reported dislike of the taste, further suggesting that a larger selection of flavours could improve compliance. Furthermore, minor side-effects were reported in relation S-ONS intake, such as decreased appetite, nausea and weight gain. Since it was not possible to change the study drink, several other strategies were made to increase intake and increase the difference in the energy and protein intake between groups. After discussion with experienced dieticians from our department we developed information material regarding the S-ONS. We changed the procedure to recommend the participants to consume one of the drinks in the morning and the other in the evening, rather than at times of day most suitable for them. We expected that this could help with remembering to consume the S-ONS. The information material highlights the S-ONS as a treatment which is important for the rehabilitation, particularly in preserving and supporting muscle strength. These recommendations were supported through the weekly follow-up conversations.

Tree out of 11 participants in the control group consumed ONS. Still, this might add significantly to the average intake of energy and protein and is relevant to consider also in a future trial where the aim is for the intervention group to achieve a higher intake of energy and protein than the control group.

### Considerations to improve data collection

Feasibility of the daily drink diary was relatively low, with only 63% of participants completing and returning it at follow-up. Despite this, it was decided to keep the diary and continue to encourage the participants to document their intake. In addition, the investigators will be more specific regarding assessing compliance by recording the reported amount of S-ONS consumed during the weekly follow-up calls.

In general, the completeness of outcome data collection was high and above 80% for most outcomes both at baseline and 12 weeks follow-up. An exception was the 30 s-CST which is planned as the primary outcome in the definitive RCT. A high percentage of the participants was unable to perform the test at baseline. However, a major advantage of the 30 s-CST compared to the Chair stand test (5-times sit-to-stand) is the possibility to have a score of zero for participants unable to rise from a chair [37]. Further, since the majority of participants were able to perform the 30 s-CST at the 12-week follow-up, it will be possible to assess an eventual clinically relevant difference in the number of stands between the two groups. The clinically relevant difference for the 30 s-CST has been found to be 2.0–2.6, when assessed in older populations with hip and knee osteoarthritis [43].

### Strength and limitations

An important strength of this trial is its inclusion of a representative population of older hip fracture patients, including very old and frail patients who are operated. This enables an evaluation of the feasibility and acceptability of study participation within this particularly vulnerable group.

As previously mentioned, the clinical context has changed since earlier studies. Particularly the widespread use of GLP-1 receptor agonists for obesity and type 2 diabetes, which can reduce appetite and food intake. In this study participants with an explicit intention to lose weight were excluded. However, patients using GLP-1 agents or other appetite-modulating drugs were not systematically identified. As these medications may influence adherence and intake, they will be documented and evaluated as potential confounders in the definitive trial.

Because of the novelty of this trial, the findings also reflect the feasibility of performing this type of trial within the context of present clinical practice, which includes fast-track surgery, shorter hospital stays, and early rehabilitation.

The conduct of this trial allows for assessment of outcome measures and methods to evaluate the feasibility for the definitive trial. Findings illustrate that it was possible to collect meaningful, patient relevant study outcomes including physical function and QoL. Further, it allows for possible adjustments to refine and improve the recruitment, inclusion strategies, and the data collection process.

A limitation of this trial is that these findings are based only on data from a relatively small sample size of 21 participants, which may limit generalizability. The results still provide valuable preliminary insights to include in the future conduct of the trial.

The approach and methods used in this trial were feasible with sufficient eligibility criteria, recruitment rate, retention in the study and completeness of outcome measures. Compliance was lower than expected and strategies to improve adherence have been identified and will be implemented in the future definitive trial. Given the findings of this feasibility trial and previous reports of challenges with compliance, future research should explore implementation strategies to enhance adherence to ONS. Qualitative insights into reasons for adherence or non-adherence will be included in the definitive trial.

## Supplementary Information

Additional file 1.

## Data Availability

The datasets analyzed during the current study are not publicly available due to the continuance of the definitive trial.

## Abbreviations

ADL: Activities of daily living
BADL: Basal activities of daily living
DHA: Docosahexaenoic acid
EPA: Eicosapentaenoic acid
EQ-5D-5L: The European Quality of life-5 Dimensions-5 Levels
FRS: Functional Recovery score
HGS: Hand grip strength
HRQoL: Health-related quality of life
IADL: Instrumental activities of daily living
ONS: Oral nutritional supplementation
QoL: Quality of life
SAS: Statistical Analysis System
SNAQ: The Simplified Nutritional Appetite Questionnaire
S-ONS: Study Oral Nutritional Supplementation
VAS: Visual analogue scale
30 s-CST: 30-s chair-stand-test
